# A Systematic Review and Meta-Analysis of the Cancer-Related and Functional Outcomes of High-Intensity Focused Ultrasound, Open Radical Prostatectomy, Robot Assisted Radical Prostatectomy, and External Beam Radiation Therapy in Primary Treatment of Localized Low- or Intermediate-Risk Prostate Cancer

**DOI:** 10.5152/tud.2024.23123

**Published:** 2024-01-01

**Authors:** Bassem Toeama, Emmanuel Papadimitropoulos, Nathan Perlis, Paul Grootendorst, Bassem Hamandi

**Affiliations:** 1 University of Toronto, Faculty of Pharmacy, Ontario, Canada; 2 University of Toronto, Faculty of Medicine, Ontario, Canada

**Keywords:** Failure-free survival rate, high-intensity focused ultrasound, prostate cancer

## Abstract

**Objective:**

Prostate cancer is the second^-^ leading cause of cancer death among men. We aimed to evaluate high-intensity focused ultrasound (HIFU), open radical prostatectomy (ORP), robot-assisted radical prostatectomy (RARP), and external beam radiation therapy (RT) in the treatment of localized low- and intermediate-risk prostate cancer.

**Methods:**

We searched bibliographic databases for case–control, cohort, and randomized controlled studies. We used MeSH subject headings and free text terms for prostate cancer, HIFU, ORP, RARP, RT, failure-free survival (FFS), biochemical disease-free survival (BDFS), urinary incontinence (UI), and erectile dysfunction (ED).

**Results:**

Fourteen studies were included in the review, for a total of 34 927 participants. Among the 8 studies of HIFU as the primary treatment of localized low- and intermediate-risk prostate cancer, 4 studies reported 5-year FFS rates ranging from 67.8% to 97.8%, 3 studies reported 5-year BDFS ranging from 58% to 85.4%, 5 studies reported 1-year UI rates ranging from 0% to 6%, and 4 studies reported 1-year ED rates ranging from 11.4% to 38.7%. Furthermore, our search revealed a 5-year FFS benefit favoring ORP compared to RT, a 1-year UI rate favoring ORP compared to RARP, and a 1-year ED rate favoring ORP compared to RARP.

**Conclusion:**

Our systematic review and meta-analysis revealed lack of studies with active comparators comparing HIFU to standard of care (ORP, RARP, or RT) in primary treatment of localized low- and intermediate-risk prostate cancer. Open radical prostatectomy has favorable efficacy outcomes compared to RT, while RARP has beneficial functional outcomes compared to ORP, respectively.

**Main Points**

There are 2 prospective, multicenter, single arm cohort studies that reported High Intensity Focused Ultrasound 1-year Failure Free Survival rates of 44.9% and 67%, 4 single arm cohort studies that reported High Intensity Focused Ultrasound 5-year Failure Free Survival rates ranging from 67.8% to 97.8%, 1 prospective, multicenter, single arm cohort study that reported High Intensity Focused Ultrasound 1-year Biochemical Disease Free Survival rate of 76.6%, 3 single arm cohort studies that reported High Intensity Focused Ultrasound 5-year Biochemical Disease Free Survival rates ranging from 58% to 85.4%, 5 single arm cohort studies that reported High Intensity Focused Ultrasound 1-year Urinary Incontinence rates ranging from 0% to 6%, and 4 single arm cohort studies that reported High Intensity Focused Ultrasound 1-year Erectile Dysfunction rates ranging from 11.4% to 38.7%.
Meta-analysis of the randomized and non-randomized studies with active comparators comparing Open Radical Prostatectomy to Radiation Therapy and Open Radical Prostatectomy to Robot Assisted Radical Prostatectomy indicated a 5-year Failure Free Survival favoring Open Radical Prostatectomy compared to Radiation Therapy (OR = 3.56, 95% CI = 2.50 - 5.08), 1-year Urinary Incontinence rate favoring Open Radical Prostatectomy compared to Robot Assisted Radical Prostatectomy (OR = 0.54, 95% CI = 0.10 - 2.89), and 1-year Erectile Dysfunction rate favoring Open Radical Prostatectomy compared to Robot Assisted Radical Prostatectomy (OR = 0.46, 95% CI = 0.12 - 1.73).
There is lack of studies with active comparators comparing High Intensity Focused Ultrasound to standard of care (Open Radical Prostatectomy, Robot Assisted Radical Prostatectomy, or Radiation Therapy) in primary treatment of localized low- and intermediate-risk prostate cancer.
Open Radical Prostatectomy has favorable efficacy outcomes compared to Radiation Therapy, while Robot Assisted Radical Prostatectomy has beneficial functional outcomes compared to Open Radical Prostatectomy, respectively.

## Introduction

### Rationale

Prostate cancer is the most frequently diagnosed male cancer and the third leading cause of death from cancer among Canadian men.^1^ In Canada, 1 in 7 men will have prostate cancer, and 1 in 27 will die of it.^2^ There are different stages of prostate cancer that range from localized through locally advanced to advanced. Localized prostate cancer is confined to the prostate gland and does not grow into nearby tissues with clinical tumor node metastasis (TNM) stages cT1-T2 N0 M0 at presentation.^3^ Localized prostate cancer is further classified into low-, intermediate-, and high-risk groups of recurrence following radical treatment according to pretreatment variables of prostate-specific antigen (PSA), Gleason score, and clinical T stage. The low-risk group has pretreatment variables of PSA <10 ng/mL, Gleason score ≤6, and clinical T stages cT1c-T2a, while the intermediate-risk group has pretreatment variables of PSA 10-20 ng/mL, Gleason score 7, or clinical T stage cT2b.^4^ According to the Canadian Cancer Society (CCS) guidelines, open radical prostatectomy (ORP), robot-assisted radical prostatectomy (RARP), external beam radiation therapy (RT), and active surveillance are the main lines of primary treatment for localized low- and intermediate-risk prostate cancer.^5^ In the UK, the Urological Cancer Care Pathway Development Group of Aberdeen recommended ablative focal therapies as alternative strategies for treating localized low- and intermediate-risk prostate cancer.^6^ Ablative focal therapies include brachytherapy, cryotherapy, high-intensity focused ultrasound (HIFU), laser therapy, radiofrequency ablation, and photodynamic therapy. The European Association of Urology (EUA) has strongly recommended HIFU within clinical trials or registries for treatment of localized intermediate-risk prostate cancer, and the USA FDA approved HIFU for treatment of localized low- and intermediate-risk prostate cancer in October 2015.^7^

#### Objectives

We aimed to evaluate the cancer-related and functional outcomes, including the 1-, 3-, and 5-year failure-free survival (FFS) rates, 1-, 3-, and 5-year biochemical disease-free survival (BDFS) rates, 1-year urinary incontinence (UI) rate, and 1-year erectile dysfunction (ED) rate, of HIFU, ORP, RARP, and RT in primary treatment of localized low- and intermediate-risk prostate cancer.

## Material and Methods

The introduction, methods, results, and discussion sections were performed following the Cochrane Handbook for Systematic Reviews of Interventions and reported according to the Preferred Reporting Items for Systematic Reviews and Meta-Analyses (PRISMA) 27-item checklist.^8,9^ The PRISMA 27-item checklist is presented in the supplementary materials.

### Eligibility Criteria

The filters used in the systematic review were humans, males, age ≥ 18 years, and clinical trials. The eligible clinical trials included case–control, cohort, and randomized controlled studies. Participants in the eligible clinical trials were male patients ≥ 18 years with histologically confirmed adenocarcinoma of the prostate. Screened studies were included if the participants had localized prostate cancer TNM stage cT1-T2 N0 M0 and low- or intermediate-risk of recurrence with pretreatment variables of PSA ≤20 ng/mL and Gleason score ≤7, the participants had not received any previous treatment for prostate cancer (including hormonal therapy, radiation therapy, surgery, or chemotherapy), and the participants were candidates for primary treatment with HIFU, ORP, RARP, or RT. Screened studies were excluded if the participants had locally advanced or metastatic prostate cancer TNM stage cT3 ± N1 ± M1 or the participants had a high risk of recurrence with pretreatment variables of PSA >20 ng/mL ± Gleason score >7, or the participants had received previous treatment for prostate cancer (including surgery, hormonal therapy, radiation therapy, or chemotherapy).

### Information Sources

We searched bibliographic databases from inception through August 31, 2021, for all relevant published case–control, cohort, and randomized controlled studies that investigated the cancer-related and functional outcomes of HIFU, ORP, RARP, and RT as primary lines of treatment for localized low- and intermediate-risk prostate cancer. Medline, Embase, and Cochrane databases were accessed via PubMed, Ovid, and Wiley interfaces, respectively.

### Search Strategy

The Patient, Intervention, Comparator, Outcome, Time, Study Type (PICOTS) model was applied to identify key topics that determined the systematic review search strategy. Patients had prostate cancer, the intervention was HIFU, ORP, RARP, or RT, the comparator was HIFU, ORP, RARP, RT, or no comparator, the outcomes were FFS rate, BDFS rate, UI rate, and ED rate, the time of data collection was at 1, 3, and 5 years for the FFS and BDFS rates and at 1 year for the UI and ED rates, and the study type was case–control, cohort, and randomized controlled studies. We mapped MeSH-controlled terms (subject headings) and searched keyword terms (synonyms) for the PICOTS components. We used truncation and search tags for the controlled terms (subject headings) and the keyword terms (synonyms), grouped controlled and keyword terms together using the Boolean AND or OR, and considered how final sets will be grouped together using the Boolean AND or OR [Supplement 1: https://docs.google.com/document/d/17mLpMjjLztTaZ8fTAX6Y5zILTilYTRFBfv3dnT1m1rA/edit?usp=sharing].

### Selection and Data Collection Process

The systematic review was performed by 2 independent reviewers, where studies were identified via databases, deduplicated, filtered, screened against inclusion and exclusion criteria, assessed for eligibility, and selected for the systematic review.

### Data Items

The studies selected for the systematic review evaluated cancer-related and functional outcomes, including the 1-, 3-, and 5-year FFS rates, 1-, 3-, and 5-year BDFS rates, 1-year UI rate, and 1-year ED rate of HIFU, ORP, RARP, and RT in primary treatment of localized low- and intermediate-risk prostate cancer. Failure-free survival is either the time from randomization or the time from primary treatment to the first of the following events: progression either locally, in lymph nodes, or in distant metastases, or death from prostate cancer. Failure-free survival includes disease-free survival, progression-free survival, metastasis-free survival, relapse-free survival, or cancer-specific survival. Biochemical disease-free survival is either the time from randomization or the time from primary treatment to PSA level ≥ 0.2 ng/mL following radical prostatectomy, PSA level ≥ 2 ng/mL above nadir PSA following radiation therapy, or PSA level ≥ 1.2 ng/mL above nadir following HIFU.^10^ Biochemical disease-free survival is also known as biochemical relapse-free survival, biochemical recurrence-free survival, biochemical failure-free survival, or PSA-failure-free survival. Urinary incontinence is the leaking of urine following HIFU, ORP, RARP, or RT, requiring the use of ≥ 1 pad per day. Urinary incontinence is also known as stress incontinence, urge incontinence, overflow incontinence, or mixed incontinence. Erectile dysfunction is the inability to achieve and sustain an erection following HIFU, ORP, RARP, or RT, sufficient for sexual intercourse. Erectile dysfunction is also known as impotence or sexual dysfunction.

### Study Risk of Bias Assessment and Reporting Bias Assessment

Reviewers followed the Newcastle–Ottawa Scale (NOS) for cohort studies, the revised Cochrane Risk of Bias tool for randomized trials (ROB 2), and the Risk of Bias in Non-randomized Studies – of Interventions (ROBINS-I) tool to identify studies at high risk of bias, and differences were resolved through discussion.

### Synthesis Methods and Effect Measures

Descriptive characteristics, including anthropometric data (weight, height, and body mass index), sociodemographic data (age, race, and ethnicity), and medical history and comorbidity data such as hypertension, diabetes, and heart disease have been balanced in some studies with active comparators and imbalanced in others. The prostate cancer characteristics, such as the PSA, Gleason score, and clinical T stage, have been balanced in studies with active comparators. The assessment outcomes of the studies lacking active comparators were analyzed with Excel’s Data Analysis Toolpak using maximum likelihood estimation and reported as point estimate (ˆ*p*), while the assessment outcomes of the randomized and non-randomized studies with active comparators were analyzed with Cochrane Tools using a random-effects model with Mantel–Haenszel (M-H) estimator for Tau^2^ statistic and reported as odds ratio (OR). Quantitative (continuous) data were represented as means and SDs, while qualitative (categorical) data were represented as frequencies and percentages.^11,12^

### Certainty Assessment

The confidence interval was set to 95% by the normal approximation method, and the margin of error accepted was set to 5%.

## Results

### Study Selection

**Figure 1.**
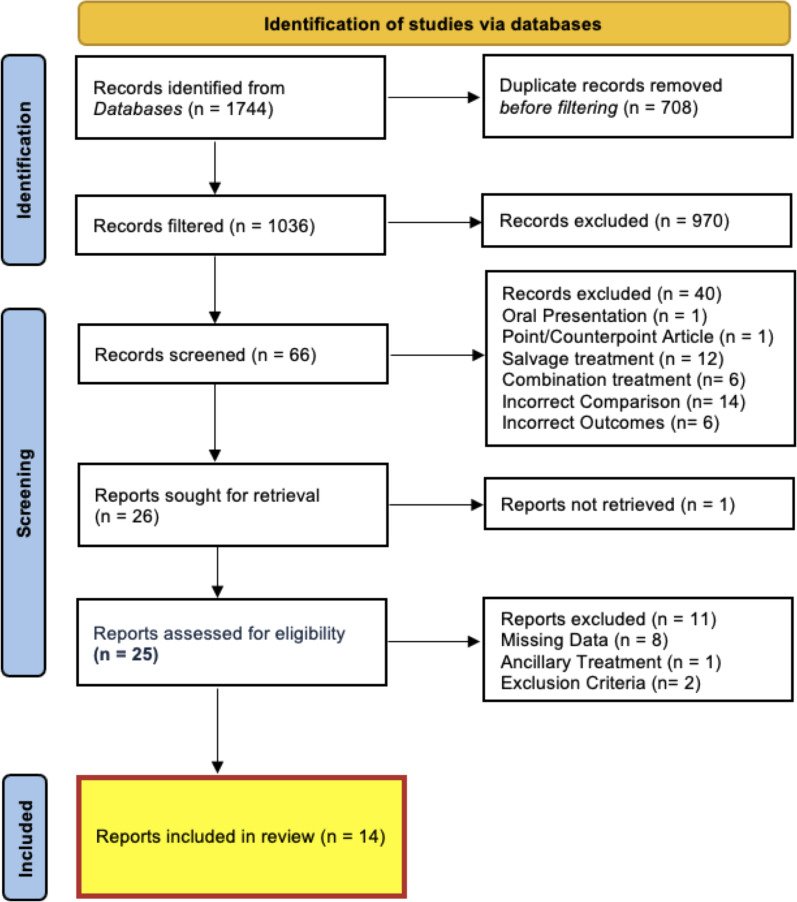
PRISMA flow chart showing the identification of the systematic review studies via the databases.

A total of 1744 studies were identified via databases. Seven hundred eight duplicate studies were detected. After deduplication, 1036 unique studies were cross-checked against the filters. Nine hundred seventy studies were removed because they did not have the sought filters. The remaining 66 studies were reviewed against the inclusion/exclusion criteria. Forty studies did not meet the inclusion/exclusion criteria, and the remaining 26 studies were sought for retrieval. One study was not retrieved, and out of the 25 studies that were retrieved, 11 reports were excluded because of missing data, ancillary treatment, and exclusion criteria. Only 14 studies were included in the systematic review (1 study evaluated HIFU for focal ablation of the prostate, 3 studies evaluated HIFU for hemiablation of the prostate, 4 studies evaluated HIFU for whole-gland ablation of the prostate, 2 studies compared ORP vs. RARP, 3 studies compared ORP vs. RT, and 1 study evaluated RT of the prostate). These studies used a total of 34 927 participants. The PRISMA flow chart reflecting the identification of studies via databases, deduplication, screening, and assessment for eligibility is presented in Figure 1.

### Study Characteristics

The key features, descriptive characteristics, and prostate cancer risk characteristics of the included studies are presented in Tables 1 and 2, respectively.

### Risk of Bias in Studies and Reporting Biases

We examined the quality of evidence for each outcome according to NOS for cohort studies, the ROB 2 tool for randomized trials, and the ROBINS-I tool for non-randomized studies. The overall quality of evidence for each outcome was determined to be high, moderate, low, or very low, as presented in Supplement 2.^13^

## Results of Individual Studies, Results of Syntheses, and Certainty of Evidence

### Failure-Free Survival

**Figure 2.**
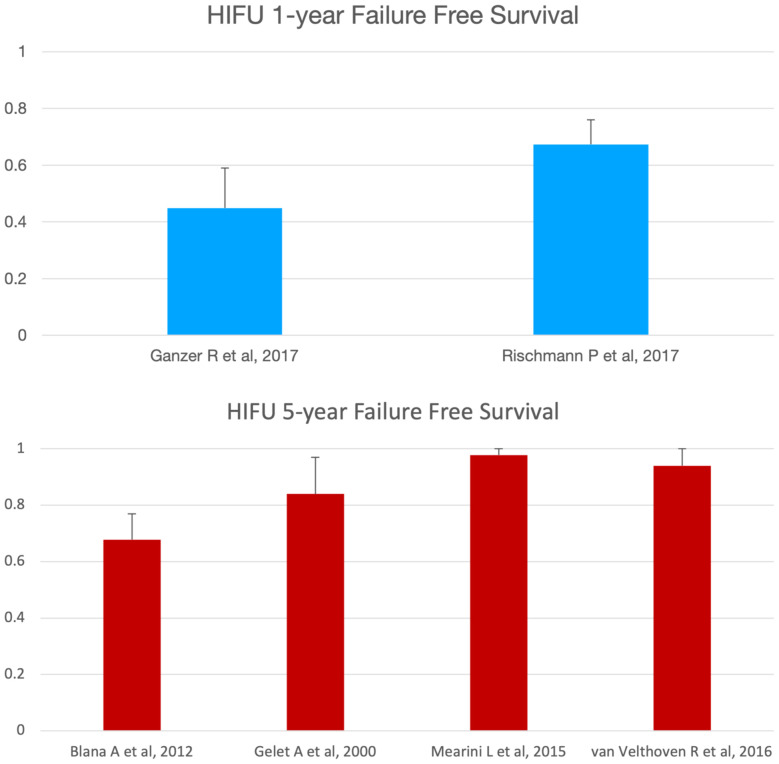
Bar charts showing the 1-year and 5-year failure-free survival rates of HIFU studies lacking active comparators with upper 95% CI. The definition of failure-free survival is not consistent among the HIFU studies lacking active comparators, the method used for calculating the point estimate (ˆ*p*) is the maximum likelihood estimation, and the method used for calculating 95% CI is the normal approximation method. HIFU, high-intensity focused ultrasound.

**Figure 3.**
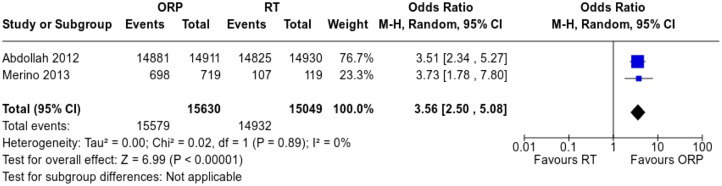
Forrest plot showing the odds ratio of ORP 5-year failure-free survival vs. RT 5-year failure-free survival with 95% CI. The definition of failure-free survival is not consistent among the ORP vs. RT double-arm trials, the descriptive characteristics and the prostate cancer risk characteristics were balanced in Abdollah et al study, the prostate cancer risk characteristics were balanced and the descriptive characteristics were unbalanced in the Merino et al study, the method used for calculating the Tau^2^ statistic is the Mantel–Haenszel estimation, and the method used for calculating heterogeneity is the random-effects meta-analysis model. ORP, open radical prostatectomy; RT, radiation therapy.

Among the 8 studies of HIFU as the primary treatment of localized low- and intermediate-risk prostate cancer, 2 studies reported 1-year FFS survival rates of 44.9% and 67% (ˆ*p* = 0.449-0.673; 95% CI, 0.31-0.76), and 4 studies reported 5-year FFS rates ranging from 67.8% to 97.8% (ˆ*p* = 0.678-0.978; 95% CI, 0.58-1) (Figure 2). In addition, our search revealed a 5-year FFS rate favoring ORP compared to RT (OR = 3.56; 95% CI, 2.50-5.08) (Figure 3).

### Biochemical Disease-Free Survival

**Figure 4.**
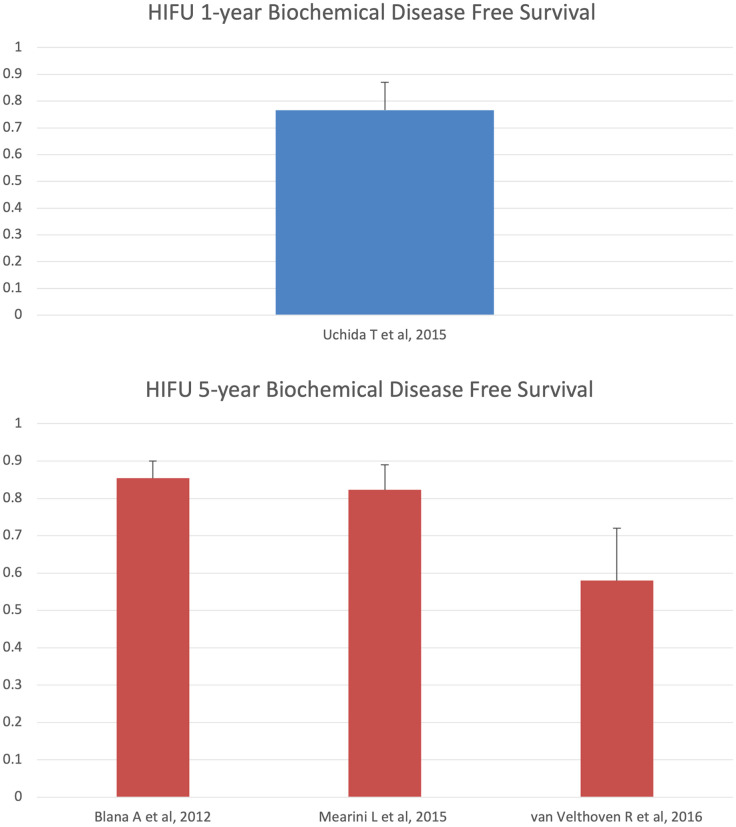
Bar charts showing the 1-year and 5-year biochemical disease-free survival rates of HIFU studies lacking active comparators with upper 95% CI. The definition of biochemical disease-free survival is not consistent among the HIFU studies lacking active comparators, the method used for calculating the point estimate (ˆ*p*) is the maximum likelihood estimation, and the method used for calculating 95% CI is the normal approximation method. HIFU, high-intensity focused ultrasound.

**Figure 5.**
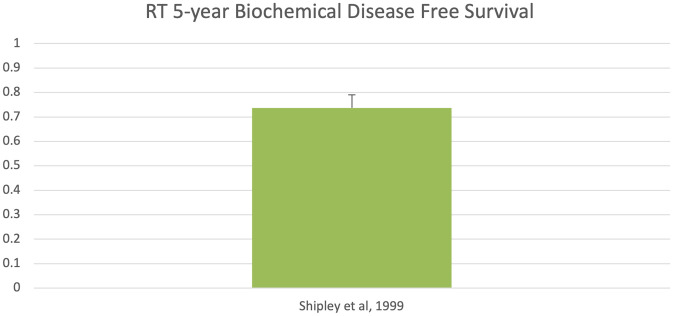
Bar chart showing the 5-year biochemical disease-free survival rate of RT study lacking active comparator with upper 95% CI. The method used for calculating the point estimate (ˆ*p*) is the maximum likelihood estimation, and the method used for calculating 95% CI is the normal approximation method. RT, radiation therapy.

**Figure 6.**
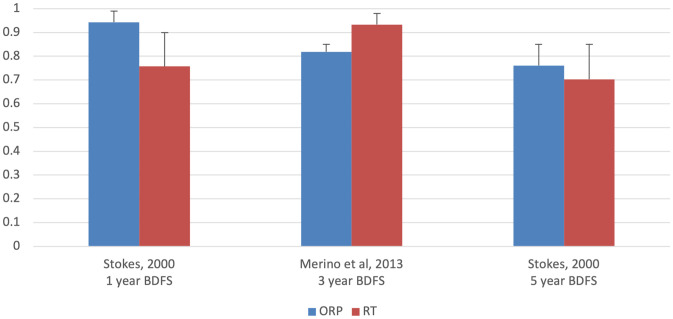
Bar chart showing the 1-year, 3-year, and 5-year biochemical disease-free survival rates of ORP vs. RT double-arm trials with upper 95% CI. The definition of biochemical disease-free survival is not consistent among the ORP vs. RT double-arm trials, the prostate cancer risk characteristics were balanced, and the descriptive characteristics were unbalanced in both Stokes et al and Merino et al studies, the method used for calculating the point estimate (ˆ*p*) is the maximum likelihood estimation, and the method used for calculating 95% CI is the normal approximation method. ORP, open radical prostatectomy; RT, radiation therapy.

Among the 8 studies of HIFU as the primary treatment of localized low- and intermediate-risk prostate cancer, 1 study reported a 1-year BDFS rate of 76.6% (ˆ*p* = 0.766; 95% CI, 0.66-0.87) and 3 studies reported a 5-year BDFS rates ranging from 58% to 85.4% (ˆ*p* = 0.58-0.854; 95% CI, 0.44-0.9) (Figure 4). In addition, our search revealed 1 RT study which reported 5-year BDFS rate of 73.7% (ˆ*p* = 0.737; 95% CI, 0.67-0.79) (Figure 5), 1 ORP vs. RT study which reported a 1-year BDFS rate of 94.3% vs. 75.7% (ˆ*p* = 0.943 vs. 0.757; 95% CI: 0.89-0.99 vs. 0.62-0.90) and 5-year BDFS rate of 76.1% vs. 70.3% (ˆ*p* = 0.761 vs. 0.703; 95% CI, 0.67-0.85 vs. 0.56-0.85), and 1 ORP vs. RT study which reported a 3-year BDFS rate of 81.9% vs. 93.3% (ˆ*p* = 0.819 vs. 0.933; 95% CI, 0.79-0.85 vs. 0.89-0.98), respectively (Figure 6).

### Urinary Incontinence

**Figure 7.**
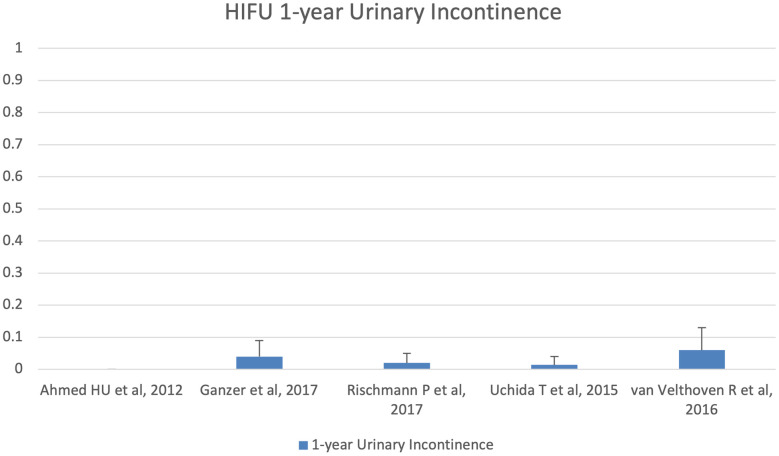
Bar chart showing the 1-year urinary incontinence rates of HIFU studies lacking active comparators with upper 95% CI. The definition of urinary incontinence is consistent among the HIFU studies lacking active comparators, the method used for calculating the point estimate (ˆ*p*) is the maximum likelihood estimation, and the method used for calculating 95% CI is the normal approximation method. HIFU, high-intensity focused ultrasound.

**Figure 8.**
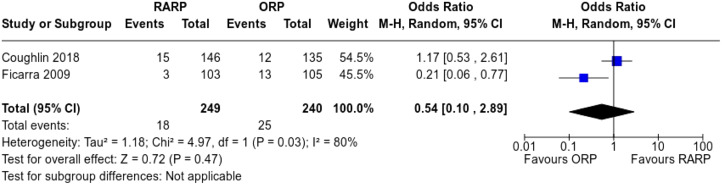
Forrest plot showing the odds ratio of ORP 1-year urinary incontinence vs. RARP 1-year urinary incontinence with 95% CI. The definition of urinary incontinence is consistent among the ORP vs. RARP double-arm trials, the descriptive characteristics and the prostate cancer risk characteristics were balanced in Coughlin et al study, the prostate cancer risk characteristics were balanced and the descriptive characteristics were unbalanced in the Ficarra et al study, the method used for calculating the Tau^2^ statistic is the Mantel–Haenszel estimation, and the method used for calculating heterogeneity is the random-effects meta-analysis model. ORP, open radical prostatectomy; RARP, robot-assisted radical prostatectomy.

Among the 8 studies of HIFU as the primary treatment of localized low- and intermediate-risk prostate cancer, 5 studies reported 1-year UI rates ranging from 0% to 6% (ˆ*p* = 0-0.06; 95% CI, 0.0-0.13) (Figure 7). In addition, our search revealed 1-year UI rate favoring ORP compared to RARP (OR = 0.54; 95% CI, 0.10-2.89) (Figure 8).

### Erectile Dysfunction

**Figure 9.**
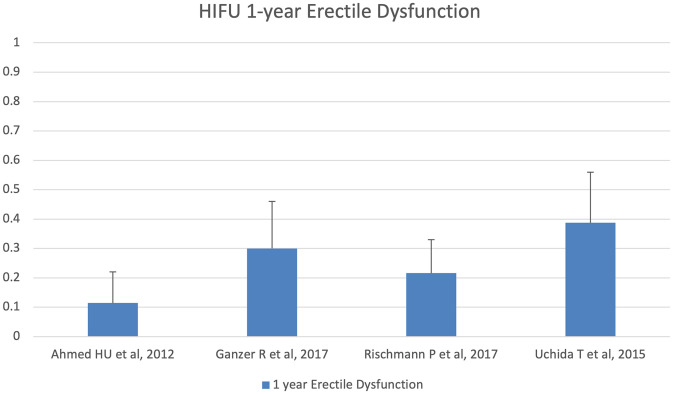
Bar chart showing the 1-year erectile dysfunction rates of HIFU studies lacking active comparators with upper 95% CI. The definition of erectile dysfunction is consistent among the HIFU studies lacking active comparators, the method used for calculating the point estimate (ˆ*p*) is the maximum likelihood estimation, and the method used for calculating 95% CI is the normal approximation method. HIFU, high-intensity focused ultrasound.

**Figure 10.**
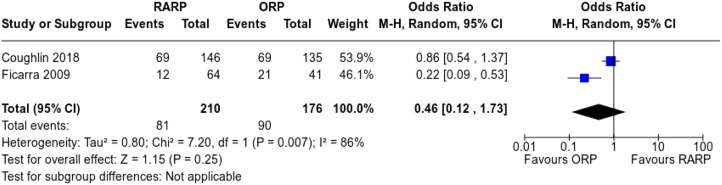
Forrest Plot showing the odds ratio of ORP 1-year erectile dysfunction vs. RARP 1-year erectile dysfunction with 95% CI. The definition of erectile dysfunction is consistent among the ORP vs. RARP double-arm trials, the descriptive characteristics and the prostate cancer risk characteristics were balanced in Coughlin et al study, the prostate cancer risk characteristics were balanced and the descriptive characteristics were unbalanced in the Ficarra et al study, the method used for calculating the Tau^2^ statistic is the Mantel–Haenszel estimation, and the method used for calculating heterogeneity is the random-effects meta-analysis model. ORP, open radical prostatectomy; RARP, robot-assisted radical prostatectomy.

Among the 8 studies of HIFU as the primary treatment of localized low- and intermediate-risk prostate cancer, 4 studies reported 1-year ED rates ranging from 11.4% to 38.7% (ˆp = 0.114-0.387; 95% CI, 0.01-0.56) (Figure 9). In addition, our search revealed 1-year ED rate favoring ORP compared to RARP (OR = 0.46; 95% CI, 0.12-1.73) (Figure 10).

## Discussion

As per the CCS and the National Comprehensive Cancer Network (NCCN) guidelines, primary treatment of localized low- and intermediate-risk prostate cancer includes active surveillance, watchful waiting, and radical local therapy (surgery and radiation therapy). Alternative focal therapy is not included in the CCS and NCCN guidelines for primary treatment of localized low- and intermediate-risk prostate cancer. The National Institute for Health and Excellence recommends using alternative focal therapy for localized prostate cancer. We conducted a systematic review and meta-analysis to compare up-to-date efficacy, safety, and functional outcomes of the second-line alternative focal therapy represented by HIFU versus up-to-date efficacy, safety, and functional outcomes of the first-line standard of care (radical local therapy) represented by ORP, RARP, and RT in the primary treatment of localized low- and intermediate-risk prostate cancer. Our systematic review showed single-arm cohort studies that lacked comparison of HIFU with the standard of care. There are 2 prospective, multicenter, single-arm cohort studies that reported HIFU 1-year FFS rates of 44.9% and 67%,^14,15^ 4 single-arm cohort studies that reported HIFU 5-year FFS rates ranging from 67.8% to 97.8%,^16-19^ 1 prospective, multicenter, single-arm cohort study that reported HIFU 1-year BDFS rate of 76.6%,^19^ 3 single-arm cohort studies that reported HIFU 5-year BDFS rates ranging from 58% to 85.4%,^16,18,19^ 5 single-arm cohort studies that reported HIFU 1-year UI rates ranging from 0% to 6%,^14,15,19,20,21^ and 4 single-arm cohort studies that reported HIFU 1-year ED rates ranging from 11.4% to 38.7%.^14,15,20,21^ In addition, our systematic review showed randomized and non-randomized studies with active comparators comparing ORP to RT and ORP to RARP. Meta-analysis indicated a 5-year FFS favoring ORP compared to RT (OR = 3.56; 95% CI, 2.50-5.08),^22,23^ 1-year UI rate favoring ORP compared to RARP (OR = 0.54; 95% CI, 0.10-2.89), and 1-year ED rate favoring ORP compared to RARP (OR = 0.46; 95% CI, 0.12-1.73).^24,25^

Limitations of our systematic review include restrictive eligibility criteria which restrained our search, decreased the number of the studies included in the systematic review, and threatened the external validity of the systematic review, heterogeneous patient populations with variations in descriptive characteristics and prostate cancer risk characteristics, lack of adjustment for the imbalanced descriptive characteristics and prostate cancer risk characteristics leading to sampling error, inconsistency of the therapeutic modality for HIFU and inconsistency of the definitions used for FFS and BDFS which might affect reliability of the results, heterogeneous results which might need further statistical tests for measuring the probability of heterogeneity, and lack of randomization in non-randomized studies with active comparators leading to selection bias.

Prostate cancer is the third leading cause of cancer death among Canadian men.^1^ Open radical prostatectomy, RARP, and RT are 3 main lines of primary treatment for localized low- and intermediate-risk prostate cancer. High-intensity focused ultrasound is an alternative treatment to ORP, RARP, and RT. Real-world evidence studies of HIFU in the primary treatment of localized low- and intermediate-risk prostate cancer are required for public funding of HIFU. There is limited published literature assessing the efficacy, safety, and functional outcomes of HIFU in the primary treatment of localized low- and intermediate-risk prostate cancer. Most of the studies that evaluated the clinical outcomes of HIFU (including efficacy, safety, and functional outcomes) were small, non-randomized, uncontrolled trials. Without sufficient comparative, well-designed studies, policymakers may face challenges in determining the appropriate place of HIFU within the existing treatment algorithm, and patients will find it difficult to engage in the shared decision-making process. Well-designed comparative studies, including large sample-sized, randomized, controlled trials, might help establish the effectiveness and safety of HIFU relative to the standard of care treatment options. In addition, HIFU technology has advanced in the past few years, healthcare providers have become more familiar and skilled with using it, and it has been recently included in the National Institute for Health and Care Excellence guidance for treatment of localized prostate cancer.^26^ The procedure of HIFU has also evolved from whole-gland treatment to focal ablation, and as a result, the adverse events of HIFU have become fewer, and its advantages as organ preservation and short recovery time have gained significance. This might be an add-on supporting clinical decision-making, incorporation into guidelines, and reimbursement for this novel treatment modality.

Our systematic review and meta-analysis revealed a lack of studies with active comparators comparing HIFU to standard of care (ORP, RARP, or RT) in the primary treatment of localized low- and intermediate-risk prostate cancer. Open radical prostatectomy has a favorable efficacy outcomes profile compared to RT, while RARP has a beneficial functional outcomes profile compared to ORP, respectively. Given the burden of prostate cancer, more studies with active comparators comparing HIFU to standard of care (ORP, RARP, or RT) in the primary treatment of localized low- and intermediate-risk prostate cancer should be prioritized for future research. This will generate less heterogeneous data and draw more robust evidence for clinical decision-making, incorporation into guidelines, and reimbursement decisions.

### Registration and Protocol Number

Our systematic review protocol has been registered in the International Prospective Register of Systematic reviews, registration number CRD 420337, and can be accessed at https://www.crd.york.ac.uk/PROSPERO/display_record.php?ID=CRD42016037337.

## Figures and Tables

**Table 1.** Characteristics of the Studies Included in the Systematic Review

Country	Study/Reference	Study Period	Study Sample	Study Design	Study Primary Endpoints
USA	Comparison of mortality outcomes after radical prostatectomy versus radiotherapy in patients with localized prostate cancer: A population-based analysis/ Abdollah et al, 2012	1992-2005	Double-arm trial of 141 155 patients. 22 144 (32.2%) patients were treated with radical prostatectomy, versus 46 521 (57.8%) patients with radiotherapy. RP patients harbored a more advanced grade at presentation (Gleason score 2-5: 4.9 vs. 5.5%, 6-7: 68.2 vs. 67.8%, and 8-10: 26.9 vs. 26.7%, *P* < .001) relative to RT patients.	Propensity Score Matched Cohort, where patients were stratified according to prostate cancer risk characteristics (high-risk: Gleason score 8-10 vs. low-intermediate risk: all other patients), baseline Charlson Comorbidity Index, and age.	Disease-free survival rates at 1, 3, 5, and 10 years.
UK	Focal therapy for localized unifocal and multifocal prostate cancer: a prospective development study/ Ahmed HU et al, 2012	2007-2010	Single-arm cohort of 41 patients (who represent low- and intermediate-risk patients) was included in the analysis.	Two-center, prospective development study with a median follow-up of 12 months and a mean age of 63 years.	Urinary symptoms and erectile function were assessed using patient questionnaires.
Nine European Centers	Complete high-intensity focused ultrasound in prostate cancer: outcome from the @-Registry/ Blana A et al, 2012	1994-2009	Single-arm cohort of 356 patients. 301 patients (84.5%), who represent low- (44.9%) and intermediate-risk (39.6%) patients, was included in the analysis.	Multicenter retrospective – prospective with a median follow-up of 2.8 years Mean age was 69.6 ± 7.2 years.	Disease-free survival rates at 1, 3, and 5 years. BDFS rates at 1, 3, and 5 years.
Australia	Robot-assisted laparoscopic prostatectomy versus open radical retropubic prostatectomy: 24-month outcomes from a randomized controlled study/ Coughlin et al, 2018	2010-2014	Double-arm trial of 326 patients. 163 (50%) patients were assigned to robot-assisted radical prostatectomy vs. 163 (50%) patients with open radical retropubic prostatectomy.	Two-group randomized controlled phase 3 trial with data from 6 months, 12 months, and 24 months. Patients were included if they were diagnosed with clinically localized prostate cancer, had chosen surgery as their treatment approach, and were aged between 35 and 70 years.	Urinary function (urinary domain of EPIC), sexual function (sexual domain of the EPIC and IIEF) assessed at 6 months, 12 months, and 24 months, and oncological outcomes (as assessed by biochemical recurrence [PSA ≥ 0·2 ng/mL] and imaging evidence of progression during the 24 months.
Italy	A prospective, non-randomized trial comparing robot-assisted laparoscopic and retropubic radical prostatectomy in 1 European institution/Ficarra et al, 2009	2006-2007	Double-arm trial of 326 patients. 103 patients were treated with robot-assisted radical prostatectomy (RARP) vs. 105 patients treated with ORP.	Non-randomized, prospective comparative study. Median age was 61 years for the RARP group vs. 65 years for the ORP group.	Urinary continence and erectile function at the 12-month follow-up.
Germany	Prospective multicenter phase II study on focal therapy (Hemiablation) of the prostate with high-intensity focused ultrasound/Ganzer R et al, 2017	2013-2016	Single-arm cohort of 54 unilateral low/intermediate-risk prostate cancer patients and 51 patients (94.4%) who completed the study were included in the analysis.	Multicenter prospective with mean follow-up of 17.4 ± 4.5 months. Mean age was 63.4 ± 8.3 years.	Disease-free survival rate at 1 year. Erectile dysfunction and urinary incontinence rates at baseline, 3, 6, 9, and 12 months.
France	Transrectal high-intensity focused ultrasound: minimally invasive therapy of localized prostate cancer/ Gelet A et al, 2000	1992-1999	Single-arm cohort of 82 biopsy-proven localized (stage T1-T2) cancer patients were not suitable candidates for radical surgery.	Single-center retrospective – prospective with a mean follow-up of 17.6 months (range: 3-68 months) and mean age of 71 ± 5.7 years (range: 60-86 years).	Disease-free survival rate at 5 years after transrectal HIFU ablation.
Italy	High-intensity focused ultrasound for the treatment of prostate cancer: A prospective trial with long-term follow-up. Mearini L et al, 2015	2004-2007	Single-arm cohort of 163 consecutive men with T1–T3 N0 M0 prostate cancer. About 127 patients (49.1%), who represent low (49.1%) and intermediate risk (28.8%) patients, were included in the analysis.	Single-center prospective study. Of the 163 patients, 160 (98.2%) were followed up for a median time of 71.5 months (66.1-73.2 months) and 3 patients were lost to follow-up.	Disease-free survival rate at 5 and 8 years.
Chile	Intensity-modulated radiotherapy versus radical prostatectomy in patients with localized prostate cancer: long-term follow-up/ Merino et al, 2013	1999-2010	Double-arm trial of 1200 patients. 993 patients were treated with ORP vs. 207 patients treated with RT. Patients were stratified according to the D’Amico classification.	Single-center retrospective with median follow-up of 91.7 months for the ORP group and 76 months for the RT group. The ORP group was significantly younger than the RT group, with average ages of 63 and 70 years, respectively.	Disease-free survival rate at 5 years.
France	Focal high-intensity focused ultrasound of unilateral localized prostate cancer: A prospective multicentric hemiablation study of 111 patients/ Rischmann P et al, 2017	2009-2015	Single-arm cohort of 111 treatment-naive patients with T1/T2 clinical stage prostate cancer. No patient was lost to follow-up.	Multicenter prospective with mean follow-up of 30.4 months. The mean age was 64.8 ± 6.2 years.	Disease-free survival rate and erectile dysfunction rate at 1 year.
USA	Radiation therapy for clinically localized prostate cancer. A multi-institutional pooled analysis/ Shipley et al, 1999	1988-1995	Single-arm cohort of 1765 patients with T1b, T1c, and T2 prostate cancer. Patients were stratified according to the Gleason score.	Multicenter retrospective, non-randomized, multi-institutional pooled analysis with a median follow-up of 4.1 years and a median age of 71 years.	Disease-free survival rate at 5 years.
USA	Comparison of biochemical disease-free survival of patients with localized carcinoma of the prostate undergoing radical prostatectomy, transperineal ultrasound-guided radioactive seed implantation, or definitive external beam irradiation/ Stokes, 2000	1988-1994	Triple-arm trial of 540 patients. 132 patients were treated with RT versus 186 patients treated with radioactive seed implantation vs. 222 patients treated with ORP.	125 patients (23.1%), who represent low- and intermediate-risk patients treated with RT and ORP, were included in the analysis.	BDFS at 1 and 5 years.
Japan	Transrectal high-intensity focused ultrasound in the treatment of localized prostate cancer: A multicenter study/ Uchida T et al, 2005	Not mentioned	Single-arm cohort of 72 consecutive patients with stage Tlc-2 NO MO prostate cancer.	Multicenter prospective with median follow-up of 14 months. The median age was 72 years.	BDFS, UI, and ED at 1 year.
Belgium	A prospective clinical trial of HIFU hemiablation for clinically localized prostate cancer/ van Velthoven R et al, 2016	Not mentioned	Single-arm cohort of 50 consecutive patients with 48% as low- and 52% as intermediate-risk prostate cancer according to the D’Amico classification.	Single-center prospective phase IIa feasibility. Study with median follow-up of 39.5 months. Median age was 73 years.	5-year actuarial metastases-free survival, BDFS, cancer-specific survival, and overall survival rates.

BMI, Body mass index; HIFU, high-intensity focused ultrasound; ORP, open radical prostatectomy, PSA: prostate specific antigen; RARP, robot assisted radical prostatectomy; RT, radiation therapy.

**Table 2.** Descriptive Characteristics and Prostate Cancer Risk Characteristics of the Studies included in the Systematic Review

	**Study Design**	**Number of Patients**	**Age**	**PSA (ng/mL)**	**Follow-up (Months)**
	**Number of Cohorts**		**Mean/Median** **(± SD/range)**	**Mean/Median** **(± SD/range)**	**Mean/Median** **(± SD/range)**
**HIFU outcome**3					
1-year FFS	Single prospective cohort	165	63.4 ± 8.3	6.2 ± 2.1	17.41 ± 4.5
	2		64.8 ± 6.2	6.2 ± 2.5	30.4 ± 14.1
5-year FFS	Single retrospective-prospective cohort	438	69.6 ± 7.2	6.83 (0.12-58)	33.6
	2		71 ± 5.7	8.11 ± 4.64	17.6 (3-68.5)
5-year FFS	Single prospective cohort	213	72 (68-75)	7.3 (5.2-10)	71.5 (66.1-73.2)
	2		74 (70-77)	6.3 (3.9-8.3)	34 (13-58)
1-year BDFS	Single prospective cohort	72	72 (45-79)	8.10 (2.1-19.8)	14 (2-24)
	1				
5-year BDFS	Single retrospective-prospective cohort	356	69.6 ± 7.2	6.83 (0.12-58)	33.6
	1				
5-year BDFS	Single prospective cohort	213	72 (68-75)	7.3 (5.2-10)	71.5 (66.1-73.2)
	2		74 (70-77)	6.3 (3.9-8.3)	34 (13-58)
1-year UI	Single prospective cohort	328	63 (58-66)	6.6 (5.4-7.7)	12 ± 0.0
			63.4 ± 8.3	6.2 ± 2.1	17.41 ± 4.5
			64.8 ± 6.2	6.2 ± 2.5	30.4 ± 14.1
	5				
			72 (45-79)	8.10 (2.1-19.8)	14 (2-24)
			74 (70-77)	6.3 (3.9-8.3)	34 (13-58)
1-year ED	Single prospective cohort	278	63 (58-66)	6.6 (5.4-7.7)	12 ± 0.0
			63.4 ± 8.3	6.2 ± 2.1	17.41 ± 4.5
	4		64.8 ± 6.2	6.2 ± 2.5	30.4 ± 14.1
			72 (45-79)	8.10 (2.1-19.8)	14 (2-24)
**ORP outcome**3					
5-year FFS	Non-randomized propensity score-matched double arm	22 144	3	3	3
	1				
5-year FFS	Non-randomized retrospective double arm	993	63 (62.6-63.5)	9.8 (9.1-10.5)	91.7
	1				
1-year BDFS	Non-randomized retrospective double arm	222	66 (43-79)	12.78 (0.4-171.6)	72 (24-120)
	1				
3-year BDFS	Non-randomized retrospective double arm	993	63 (62.6-63.5)	9.8 (9.1-10.5)	91.7
	1				
5-year BDFS	Non-randomized retrospective double arm	222	66 (43-79)	12.78 (0.4-171.6)	73 (24-120)
	1				
1-year UI	Randomized controlled	163	(35-70)	3	23.93 (23.7-24.85)
	1				
1-year UI	Non-randomized prospective double arm	105	65 (61-69)	6 (5.0-10.0)	12 ± 0.0
	1				
1-year ED	Randomized controlled	163	(35-70)	3	23.93 (23.7-24.85)
	1				
1-year ED	Non-randomized prospective double arm	105	Mean (± SD)	Mean (± SD)	12 ± 0.0
	1					**RARP outcome**3					
1-year UI	Randomized controlled	163	(35-70)	3	24 (23.74-24.69)
	1				
1-year UI	Non-randomized prospective double arm	103	61 (57-67)	6.4 (4.6-9.0)	12 ± 0.0
	1				
1-year ED	Randomized controlled	163	(35-70)	3	24 (23.74-24.69)
	1				
1-year ED	Non-randomized prospective double arm	103	Mean ± SD	Mean ± SD	12 ± 0.0
	1					**RT outcome**3					
5-year FFS	Non-randomized propensity score-matched double arm	46 521	3	3	3
	1				
5-year FFS	Non-randomized retrospective double arm	207	70 (69-71)	13.6 (11.8-16.6)	76
	1				
5-year BDFS	Single retrospective cohort	1765	71	10.1 (0.2-2028.0)	49.2 (24-108)
	1				
1-year BDFS	Non-randomized retrospective double arm	132	72 (49-87)	45.4 (1.0-625.0)	75.5 (24-120)
	1				
3-year BDFS	Non-randomized retrospective double arm	207	70 (69-71)	13.6 (11.8-16.6)	76
	1				
5-year BDFS	Non-randomized retrospective double arm	132	72 (49-87)	45.4 (1.0-625.0)	75.5 (24-120)
	1				

BMI, body mass index; HIFU; high-intensity focused ultrasound; ORP, open radical prostatectomy; PSA, prostate specific antigen; RARP, robot-assisted radical prostatectomy; RT, radiation therapy .
